# Ultrasensitive interplay between ferromagnetism and superconductivity in NbGd composite thin films

**DOI:** 10.1038/srep18689

**Published:** 2016-01-04

**Authors:** Ambika Bawa, Anurag Gupta, Sandeep Singh, V.P.S. Awana, Sangeeta Sahoo

**Affiliations:** 1 Quantum Phenomena & Applications, CSIR-National Physical Laboratory, Council of Scientific and Industrial Research, Dr. K.S. Krishnan Marg, New Delhi, 110012, India; 2 Sophisticated and Analytical Instrumentation, CSIR-National Physical Laboratory, Council of Scientific and Industrial Research, Dr. K.S. Krishnan Marg, New Delhi, 110012, India

## Abstract

A model binary hybrid system composed of a randomly distributed rare-earth ferromagnetic (Gd) part embedded in an s-wave superconducting (Nb) matrix is being manufactured to study the interplay between competing superconducting and ferromagnetic order parameters. The normal metallic to superconducting phase transition appears to be very sensitive to the magnetic counterpart and the modulation of the superconducing properties follow closely to the Abrikosov-Gor’kov (AG) theory of magnetic impurity induced pair breaking mechanism. A critical concentration of Gd is obtained for the studied NbGd based composite films (CFs) above which superconductivity disappears. Besides, a magnetic ordering resembling the paramagnetic Meissner effect (PME) appears in DC magnetization measurements at temperatures close to the superconducting transition temperature. The positive magnetization related to the PME emerges upon doping Nb with Gd. The temperature dependent resistance measurements evolve in a similar fashion with the concentration of Gd as that with an external magnetic field and in both the cases, the transition curves accompany several intermediate features indicating the traces of magnetism originated either from Gd or from the external field. Finally, the signatures of magnetism appear evidently in the magnetization and transport measurements for the CFs with very low (<1 at.%) doping of Gd.

A coupled superconductor (SC) - ferromagnet (FM) hybrid system can provide a model platform to study the interaction between two fundamentally contrasting many body ground state problems in one system where the conflicts appear due to the incompatible nature of the order parameters of its individual components. One of such existing experimentally accessible electronic hybrid systems is SC-FM based composite thin film (CF) with controllable parameters tuned by its composition. For an s-wave SC is in contact with a FM as in a CF, a modulation of the superconducting order parameter is the most pronounced effect which occurs mostly due to the proximity effect at the SC-FM interfaces, exchange interaction and stray fields originated from FM^1^. In addition, varieties of other interesting physical phenomena like coexistence of magnetism and superconductivity^2^, enhancement of superconductivity due to magnetic interaction^3^,^4^, domain wall superconductivity^5^,^6^, high critical current for power electronic application^7^, significant pinning enhancement due to the integration of FM particles into the SC matrix^8^,^9^, existence of long-range spin-polarized supercurrents^10^,^11^ etc. have been observed in SC-FM based hybrid systems. Besides, being real multicomponent percolating physical systems, CFs also offer to study the fundamental aspects of electronic, transport, and magnetic properties of individual percolating networks and understand the quantum phase transitions^12^,^13^.

To construct the CFs, we employ one of the commonly explored rare-earth magnets, Gd, which has also been used to impact the superconducting properties while coupled to a superconductor^14^,^15^. As per the SC material is considered, Nb offers the highest critical temperature for an elemental superconductor. Combination of these two would possibly leads to one of the simplest selection of SC-FM based systems. Since 1960s, NbGd based hybrid systems in various geometries have been used to study the interplay between s-wave superconductivity in Nb and ferromagnetism mediated by 4f localized electrons in Gd^8^,^16^,^17^,^18^,^19^,^20^,^21^. Recently, we have demonstrated that phase slip processes can be triggered by introducing magnetic Gd particles into superconducting Nb matrices^22^. Here, we study the modulation of the superconducting properties together with an investigation for the existence of any characteristic magnetic properties in the limit of very dilute magnetic doping in NbGd based CFs.

The CFs were grown at room temperature with thicknesses in the range between 50 to 110 nm directly on Si (100) substrate. We observe a strong pair breaking effect induced by magnetic Gd and the modulation of the superconducting critical temperature T_c_ closely follows the AG theory related to the magnetic impurity induced pair breaking mechanism for an s-wave superconductor^23^. Our results clearly demonstrate that Gd is strongly detrimental to restoring the superconductivity in the CFs and a critical concentration (*c*_cr_) of about 1.3 at.% of Gd can completely destroy the superconductivity. This is in very much contrast to a recently reported result where the authors claimed to have superconductivity sustained for up to ~40 at.% of Gd incorporation with a Nb buffer layer (50 nm)^8^. To avoid any contribution from the buffer layer and hence to directly probe the superconducting properties of CFs we do not use any SC buffer layer. Furthermore, the present findings support some of the earlier works reporting no clear superconducting transitions for the CFs having Gd more than about 5 at.%^18^,^24^.

As a true interplay between SC and FM, the signatures of magnetism have been also observed by the paramagnetic Meissner effect (PME)^25^,^26^,^27^ in the temperature dependent DC magnetization measurements (M-T) for the CFs while only diamagnetic Meissner effect is observed in pure Nb films. With increasing Gd above the *c*_cr_, a paramagnetic transition occurs and the related transition temperature T_m_ behaves linearly with Gd concentration, *c*. Besides, we observe a non-trivial resistive-superconducting transition curve featured with several intermediate structures initiated and modulated by the Gd incorporation in R-T measurements. These distinct features modulate additively with the applied magnetic field. Thus even with a very dilute magnetic doping of less than 1at.%, the characteristics of magnetic properties are evident in the magnetization as well as in the transport measurements. Finally, we have constructed a compositional phase diagram where the region related to the PME is included in addition to the other phases and have established the magnetic phase diagram for varying composition.

## Results

**Figure 1:**
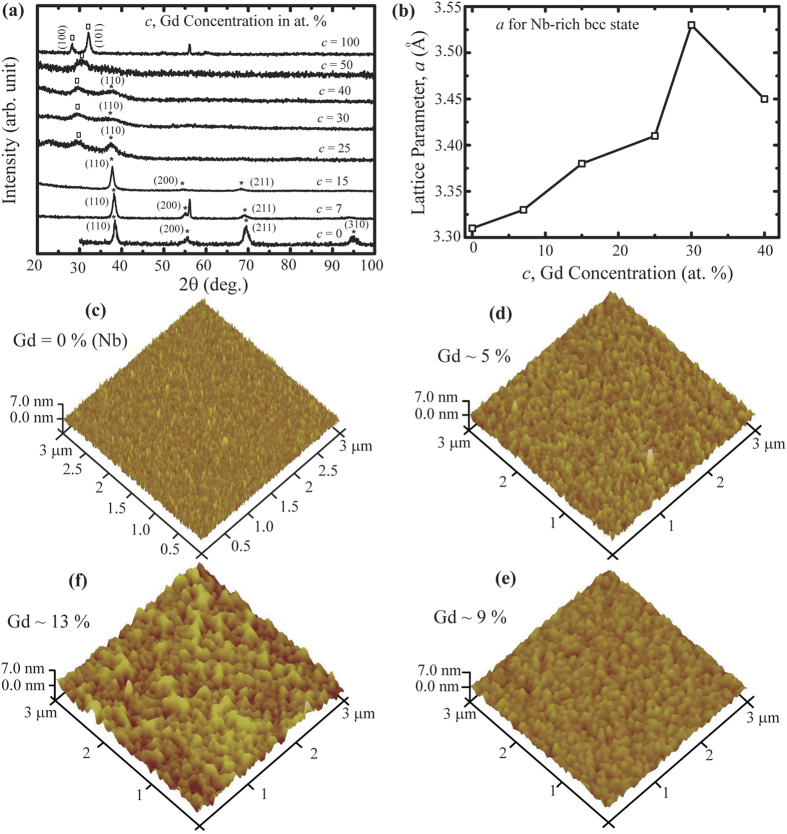
Structural and morphological characterization. (**a**) X-ray diffraction spectra of Nb-Gd composite thin film samples. The curves are shifted for the sake of clarity. The Nb-rich samples show crystalline bcc structure whereas pure Gd film shows hcp structure. Above 25 at.% of Gd a broad peak, indicated by the rectangular block, appears for all the samples. The peaks related to the Nb-rich bcc structures are marked with asterisk (‘*’) marks. (**b**) Lattice Parameter, *a* of the Nb-rich bcc state with Gd concentration *c* up to 40 at.%. (**c**–**f**) AFM images showing morphological evolution of 100 nm thick CFs with different Gd content. Surface morphology of (**c**) Nb (*c* = 0), (**d**) NbGd (*c* = 5%), (**e**) NbGd (*c* = 9%), and (**f**) NbGd (*c* = 13%).

The X-ray diffraction (XRD) patterns of as-deposited CFs having different compositions are shown in Fig. 1(a). Pure Nb film shows a body-centered cubic (bcc) structure with lattice parameter, *a* = *3.31 Å*. All the samples with relatively low *c*, the Gd concentration, up to 15 % reveal similar bcc patterns. With increasing *c*, the peak positions shift to lower 2θ values indicating an increase of the lattice parameter due to Gd inclusions. Further, for *c* ≥ 25 at.%, appearance of broad features, marked with rectangular blocks in Fig. 1, leads to either the presence of both cubic (fcc) and hexagonal phases of Gd^8^ or could be a signature of a typical amorphous solids as reported earlier^24^. Pure Gd yields a hexagonal close-packed (hcp) structure with lattice parameters, *a_hcp_* = 3.64 Å, and *c_hcp_* = 5.88 Å. Fig. 1(b) presents the variation of the lattice parameter *a* for the Nb-rich bcc state with *c* up to 40 %. The inclusion of Gd with larger metallic radius (1.87 Å) than that of Nb (1.43 Å) clearly influences and increases the lattice parameter of the Nb-rich bcc phase.

An atomic force microscope (AFM) is used to study the surface topography of the CFs. Figure 1(c–f) represents the morphological evolution of the CFs with increasing *c*. The morphology of a Nb film of about 100 nm thickness is shown in Fig. 1(c) while (d–f) of Fig. 1 represent the same for NbGd films grown in a single batch but having variation in Gd concentration. The changes in the grain size for different *c*-values are evident in the AFM images. The grain size for the Nb samples are about 20–30 nm as reported earlier also^22^ whereas, with increasing *c* the lateral size of the grains becomes larger and the grain boundaries get blunter. Further, it is visible that the grains start to merge and to form clusters type of morphology for higher concentration of Gd [Fig. 1(f)].

**Figure 2:**
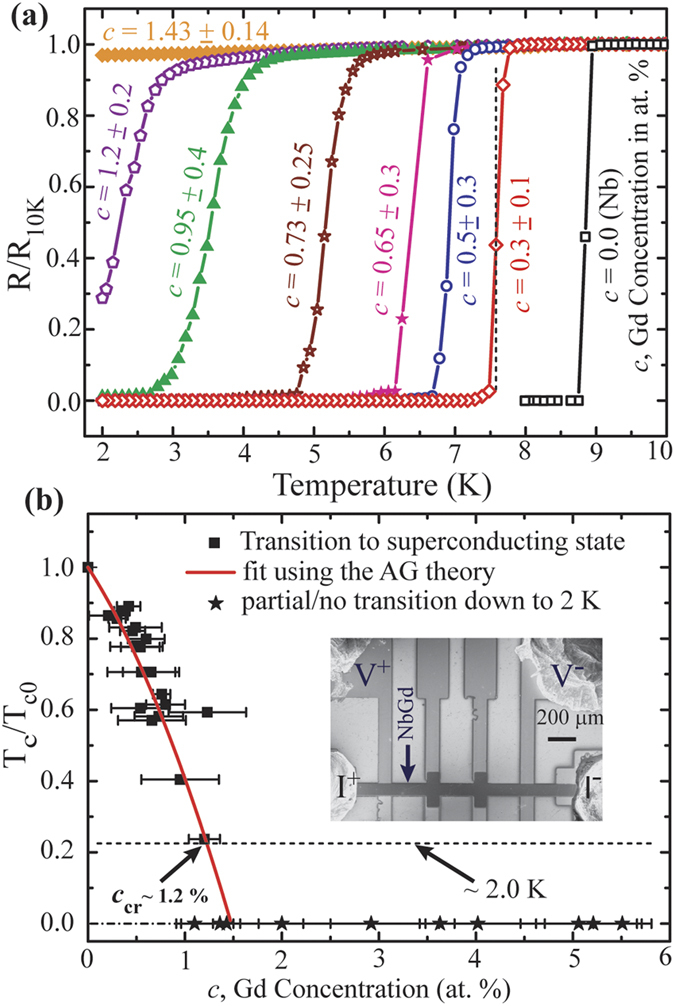
R-T measurements at zero-field. (**a**) Temperature dependent resistance measurements on Nb-Gd based CFs with different Gd concentration, *c*. The resistance is normalized by the normal state resistance measured at 10 K. The superconducting transition temperature T_c_ is defined as the temperature corresponding to the maximum value in dR/dT and is indicated by the black dashed line for a device with ‘*c*’ = 0.3 ± 0.1. (**b**) Variation of T_c_, extracted from R-T measurements, with Gd concentration, *c*. T_c_ is normalized by T_c0_, the transition temperature of pure Nb film. The scattering points represent the experimental data and the solid red curve is a fit using the Abrikosov-Gorkov (AG) theory. The intersect point between the fit and the base temperature (2 K) provides a critical value of *c*, ‘*c*_cr_’ representing the crossover from superconducting to normal state transition. The dashed lines close to 2 K represent the temperature limit in the existing measurement setup. Inset: SEM micrograph of a representative device having NbGd as the active channel connected with Au/Ti electrical contacts.

The temperature dependent resistance (R-T) measurements are carried out on CF based devices in conventional 4-terminal geometry. The Device geometry and the electrical connections are shown in the inset of Fig. 2(b). A set of normalized R-T curves with varying *c* are shown in Fig. 2(a). The resistance is normalized with the normal state resistance measured at 10 K. The superconducting transition temperature T_c_ is defined as the temperature corresponding to the maximum value of dR/dT and is shown by the dotted black vertical line for the device with *c* = 0.3 (the red curve). The black open squares in Fig. 2(a) present the R-T characteristic for a pure Nb film which shows a sharp transition with an extent of about 0.1 K in temperature and with a T_c_ ~ 8.85 K. With increasing *c*, the normal metal (NM) – superconductor transition curves move towards lower temperature. At *c* = 0.95, the resistance reduced to ~1 % of its normal state value. Further, the transitions get broader and hence an increase in transition width with increasing *c* as manifested in the R-T measurements. For *c* ≥ 1, the devices do not show a complete zero-resistance transition from its normal state down to the temperature limit of the measurement setup of ~2 K. Within this narrow range of Gd concentration we observe a crossover between superconducting to non-superconducting states as well as an obvious modulation of the superconducting transition width mediated by the magnetic contribution in the CFs.

The suppression of the T_c_ is consistent with the pair breaking picture introduced by Abrikosov and Gor’kov (AG) for an s-wave superconductor in the presence of magnetic impurities. As described by the AG theory^23^, the normalized T_c_ can be denoted as:


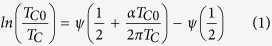


where, T_c0_ is the critical temperature of pure Nb film, 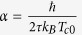
is the pair breaking parameter with *τ* as the quasi-particle lifetime controlled by the scattering from magnetic impurities . Assuming isotropic scattering in the limit of very low impurity, *τ* can be related to the impurity concentration *c* as^28^,^29^, 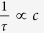
where *c* is in at.%. *ψ* is the digamma function and can be expressed as,


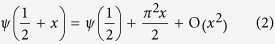


In the limit of very dilute doping with 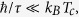 equation (1) can be rewritten as,


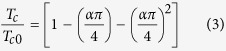


Now from equation (3) if we neglect the quadratic term for a very low range of impurity concentration, a linear dependence is expected for the critical temperature to the impurity concentration. From equation (1) we note that for a critical value of α, *α*_cr_, T_c_ vanishes. This implies that the superconductivity can sustain up to a critical impurity concentration *c*_cr_.

To envisage experimentally the dependence of the T_c_ on *c*, we have extracted T_c_ values from each R-T data for ~30 samples varying in *c*. Each T_c_ is normalized by T_c0_, the critical temperature of Nb and the normalized T_c_ is plotted against *c* in Fig. 2 (b). The black squares represent the experimental results whereas the red solid curve is a fit using equation (3). The black stars represent the T_c_ of measured samples showing partial/no transition down to 2 K. For these samples the T_c_ values are placed at the 0 K line in Fig. 2(b). The critical concentration *c*_cr_ is considered at the base temperature of the measurement setup as indicated in Fig. 2(b) and is about 1.2 at.%.

**Figure 3:**
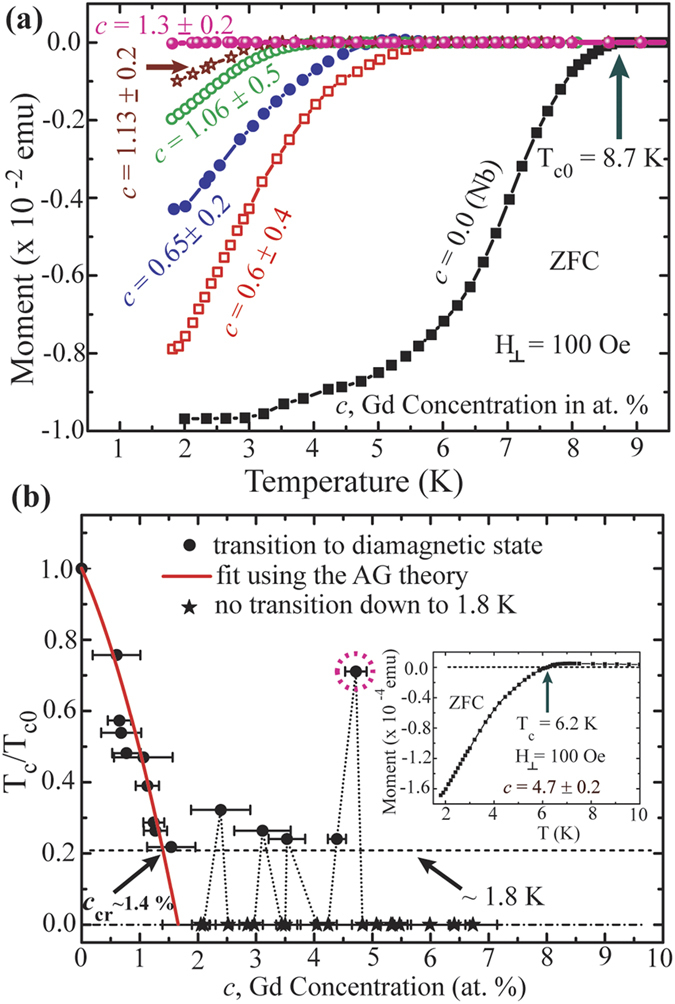
M-T measurements performed under a perpendicular field of 100 Oe in ZFC condition. (**a**) A set of M-T curves for Nb-Gd based CFs with different ‘*c*’ values. The black arrow indicates the T_c0_ value where the Nb film starts to show diamagnetic transition. (**b**) Variation of T_c_ with ‘*c*’ from the M-T measurements. T_c_ is normalized by T_c0_. The scattering points represent the experimental data and the red solid curve represents a fit using the AG theory. The crossing between the fit and the base temperature (1.8 K) provides the critical concentration value ‘*c*_cr_’ of Gd content. The dashed line close to 1.8 K represents the temperature limit of the measurement setup. The dotted curve is a guide to the eye. Inset: M-T curve of a sample represented by the pink dotted circular region in the main panel of (**b**).

As the Meissner effect is another hallmark of superconductivity we have verified the dependence of T_c_ on *c* through DC M-T measurements also. Figure 3(a) displays a set of M-T curves measured on CFs with different *c* values. The black squares represent the M-T behaviour of a Nb film with a T_c_ ~ 8.7 K as shown by the arrow in Fig. 3(a). Here, the T_c_ is defined as the temperature at which a CF starts to show negative magnetization. The measured T_c_ values for Nb from both R-T and M-T measurements are close to its bulk value (T_c0_^bulk^ = 9.2 K).

Similar to the R-T measurements, the M-T transition curves show a general trend to shift towards lower temperature with increasing *c* [Fig. 3(a)]. Likewise, we have extracted the T_c_ values from each M-T curves with different *c* and the related dependence is shown in Fig. 3(b). Up to a value of *c* ~1.4%, the variation of T_c_ on *c* follows an analogous trend as that is observed from R-T measurements in Fig. 2(b). A fit using equation (3) shown by the red curve in Fig. 3(b) follows closely to the experimental points in this range of *c*. However, above *c* ~1.4%, the diamagnetic transitions (black circles) occur irregularly for some of the samples and for the rest we observe no transition (black stars) and the related modulation in T_c_ with *c* is shown by the dotted curve in Fig. 3(b). A representative M-T curve is shown in the inset of Fig. 3(b) with *c* = 4.7% which is marked with pink dotted circular region in the main panel. Here, we observe a clear diamagnetic transition at ~6.2 K. However, the amplitude of the magnetization is ~50 times smaller than that for the samples presented in Fig. 3(a). This might be a weak transition originated locally through any percolated path but not through the whole sample and can be treated as an incomplete transition. Incomplete/partial transitions are observed in R-T measurements (Figure S3 in the supporting material) also for the devices with higher values of *c*.

It is apparent from the M-T and R-T measurements that the superconducting T_c_ collapses rapidly to the base temperature limit with increasing *c* from 0 to ~1.3 %. The close agreement between experimental results and the fit using the AG theory indicates the pair breaking mechanism induced by the magnetic doping as the origin of this suppression of superconductivity. The critical concentration *c*_cr_ of Gd provides a crossover from superconducting to normal state in these CFs. Indeed, the values of *c*_cr_ from the R-T (~1.2 % at 2 K) and M-T measurements (~1.4 % at 1.8 K) are very close.

**Figure 4:**
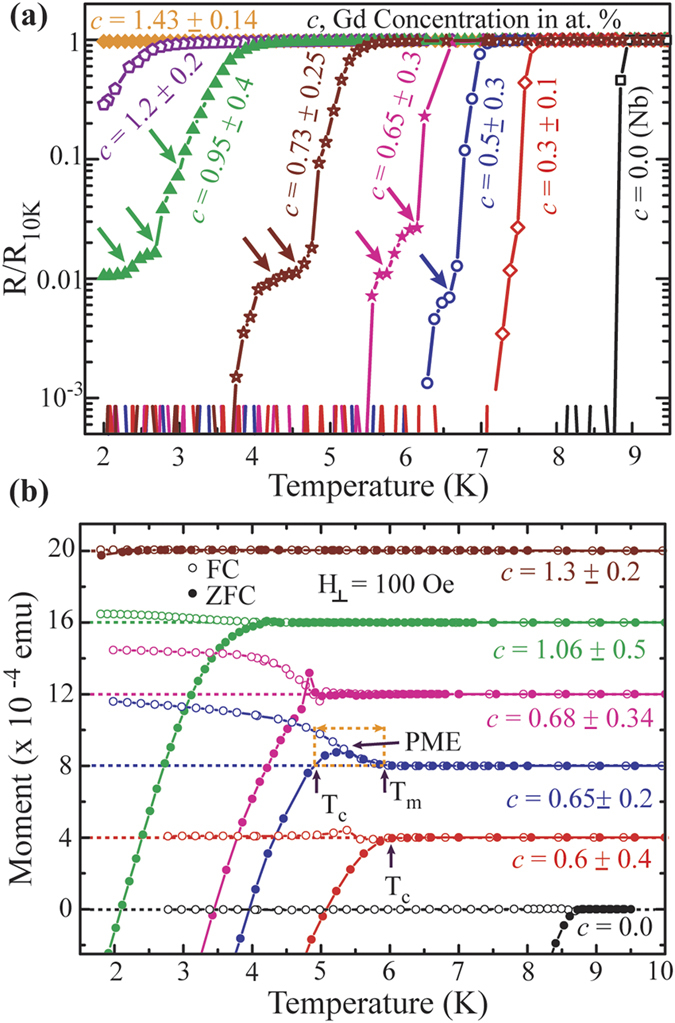
(**a**) A Semi-logarithmic presentation of R-T curves shown in Fig. 2(a). The colored arrows indicate the presence of shoulder/kink type of structures appearing for NbGd devices while they are absent in pure Nb device (**b**) Presentation of selected portion of M-T curves in FC (open circles) and ZFC (solid circles) conditions for a few representing samples close to their transition. For clarity the curves are shifted. The dotted base lines attached to each curve represent the zero- magnetization value. The orange dotted rectangular region demonstrates the paramagnetic Meissner effect (PME) where a positive magnetization is observed just before the diamagnetic transition for ZFC condition while corresponding FC curve remains positive. The superconducting critical temperature T_c_ and the temperature T_m_ related to the onset of the positive magnetization from the background are shown by the arrows.

It is clear that the magnetic doping is detrimental for the superconducting properties in the CFs and the T_c_ gets degraded with increased magnetic constituents. However, the detail mechanism behind the degradation of the superconductivity might be understood from the evolution of the SC-NM transition with the concentration of the FM part. To obtain an insight into the transition region of the R-T characteristics we display the R-T data shown in Fig. 2(a) in a semi-logarithmic scale in Fig. 4(a). As seen earlier, the Nb device adheres a sharp transition down to its complete superconducting state. In contrast, NbGd devices expose some distinct features described by kink/shoulder type of broadenings which get prominent with increasing *c*. The colored arrows in Fig. 4(a) indicate the formation of the features along with their evolution with increasing *c*. Hence, the question arises whether the featured kinks in the NbGd devices imply any trace of magnetism appearing from the Gd content.

Further, we emphasize a closer look onto the M-T data measured in both the field cooled (FC) and zero field cooled (ZFC) conditions for a few representative samples presented separately in Fig. 4(b). For clarity, we shifted the M-T curves in the y-direction with the order of increasing *c* values and the dotted baselines attached to each individual curve represent the zero-magnetization state obtained by subtracting the background magnetization. The bottom black circles represent the M-T measurements for a Nb sample with a T_c_ ~ 8.7 K. The similar behaviour is observed for the next sample (the red circles) while moving upward in Fig. 4(b) except for a very little fluctuation in the related FC data close to its T_c_. Moving to the next sample in upward direction with a little higher *c* value (blue circles), we witness a very different behaviour in ZFC M-T characteristic which shows a crossover from a positive to negative magnetization while decreasing the temperature. Unlike the former two samples, the FC data for this sample shows a strong positive magnetization and the temperature T_m_ for the onset of positive magnetization appears to be the same at which the positive magnetization appears for the ZFC M-T data. This scenario is very similar to the paramagnetic Meissner effect (PME) observed in earlier studies^30^,^31^,^32^,^33^. We highlight the region of paramagnetic to diamagnetic transition by the orange dotted rectangular region in Fig. 4(b). With increasing *c* the PME is still observed for next couple of samples in Fig. 4(b), however, the amplitude of the paramagnetic signal gets reduced and the PME disappears for the sample with *c* = 1.3 %. From the M-T measurements with further increasing *c*, we mostly observe an onset of a positive magnetization and the related temperature T_m_ increases linearly with *c*. We note that due to a smaller paramagnetic signal, T_m_ values are not distinguishable from the background in the same scale for all the individual samples presented in Fig. 4(b). However, their individual data clearly exhibit the transition to paramagnetic state over the background signal (see Figure S4 in the supporting material).

There are two most common and well accepted arguments behind the PME, namely, the d-wave symmetry of quasiparticle pairing and the flux compression^25^. The former is not relevant in our case since we observe a strong suppression of T_c_ with the concentration of magnetic impurities indicating the SC is of s-wave symmetry as described in the AG theory^23^. The latter, i.e., the magnetic flux compression is possible due to the inhomogeneity present in these CFs^34^. The PME effect has been observed in SC-FM based hybrid systems where the effect is attributed to the flux compression due to spontaneous vortex formation^35^ and also to the interplay between SC and FM in the hybrids^36^. Recently, we also have shown that the magnetic doping may lead to the vortex formation^22^. Further, according to the Ginzburg-Landau (GL) theory, the presence of vortices inside a thin superconducting sample can cause the PME by the compression of the flux trapped inside the vortices^37^,^38^. The kink types of features in R-T characteristics [Fig. 4(a)] can be of support to the picture of vortex formation^39^ and hence the flux compression. However, for better understanding of the origin of the PME one needs to study the field dependence magnetization behaviour which is out of the scope of this study. Here, we emphasize that the traces of magnetism are evident in both the DC magnetization and the R-T measurements for these NbGd based CFs with less than 1 at.% of Gd in it.

**Figure 5:**
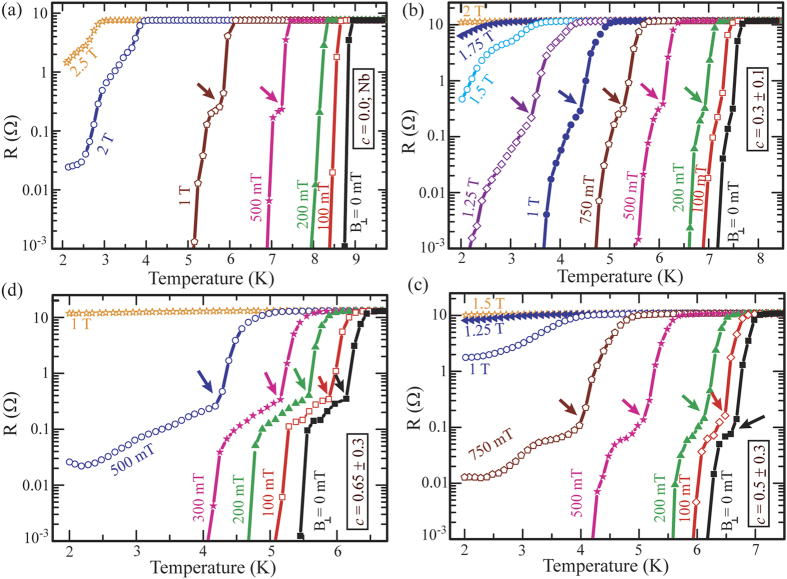
Evolution of R-T curves with magnetic field applied perpendicular to the sample plane. Field dependence of R-T characteristics for devices with (**a**) *c* = 0, i.e. for a pure Nb device, (**b**) *c* = 0.3, (**c**) *c* = 0.5, (**d**) *c* = 0.65, respectively. The formation and the positions of the shoulder-type of structures are indicated by the arrows.

For further support in favor of the signatures of the magnetic characteristics we have performed the R-T measurements under an external magnetic field applied perpendicular to the plane of the samples and their evolution with the B-field is presented in Fig. 5. Figure 5(a) shows a set of field dependent R-T measurements data for a Nb device. We observe a sharp transition from the resistive state to its superconducting state under low applied field with a reduced T_c_ for increased B-field. At relatively higher B-field, the sharpness of the transition gets distorted and it becomes wider. For example at B = 500 mT, the transition curve accompanies a kink type of structure (shown by an arrow) indicating an intermediate state possibly due to the vortex formation under the magnetic field for a type-II superconductor^39^. Thus the applied B-field above a threshold value causes similar types of features/kinks in the R-T characteristics for the Nb film as they appeared for the NbGd samples under no external field. The Field dependent R-T measurements are being carried out for NbGd composite devices also and the results for a few illustrative devices with *c* = 0.3, 0.5, 0.65 are presented in Fig. 5(b–d), respectively. In Fig. 5(b) with *c* = 0.3 and for the fields up to 100 mT, the transition curves look broader compared to that of the Nb device. However, this device with an applied field of about 200 mT facilitates the shouldering effect which was absent under no external field. At B = 200 mT, a small kink starts to appear in the transition characteristic and it gets prominent with increasing the field. Similar type of features appears for devices with higher values of *c* [Fig. 4(a)] under no external field as clearly manifested in Fig. 5(c,d). In addition to the broadening of the transition width with increasing c, the shouldering features are getting wider and stronger and they are observed to modulate in a similar fashion with the external field.

It is apparent from Fig. 4(a) and Fig. 5 that the kinks/shouldering features appeared in the R-T characteristics originate either from the magnetic particles embedded in the CF or from the external magnetic field suggesting their origin to be related with the magnetism. The features start to appear at higher field for the devices with lower *c* and *vice versa*. This is a clear indication that the applied field and the concentration of the magnetic counterpart in the CFs supplement each other. Hence it is straightforward that the magnetic component of the CFs generates an effective magnetic field which probably is originated from the exchange interaction and/or the stray field associated with the magnetic particles^39^. The magnetic contribution of Gd in the CFs is also evident by the appearance of positive magnetization in ZFC and the occurrence of the PME in the M-T measurements as shown in Fig. 4(b). Further, Nb is a type-II superconductor and the vortex states are expected to play an important role while studying the interaction between the superconductivity and the ferromagnetism in Nb-Gd based hybrid systems. The incorporation of magnetic Gd into the Nb matrix can lead to vortex state due to its internal field and also it can act as the pinning centers under an applied magnetic field. The shouldering type of features appearing in the R-T curves open up a possibility to have spontaneous vortices in the CFs having the Gd concentration above a threshold value. In this study, the features start to appear for *c* = 0.5% which could be the threshold value of *c* to observe the features at zero external B-field.

## Discussion

**Figure 6:**
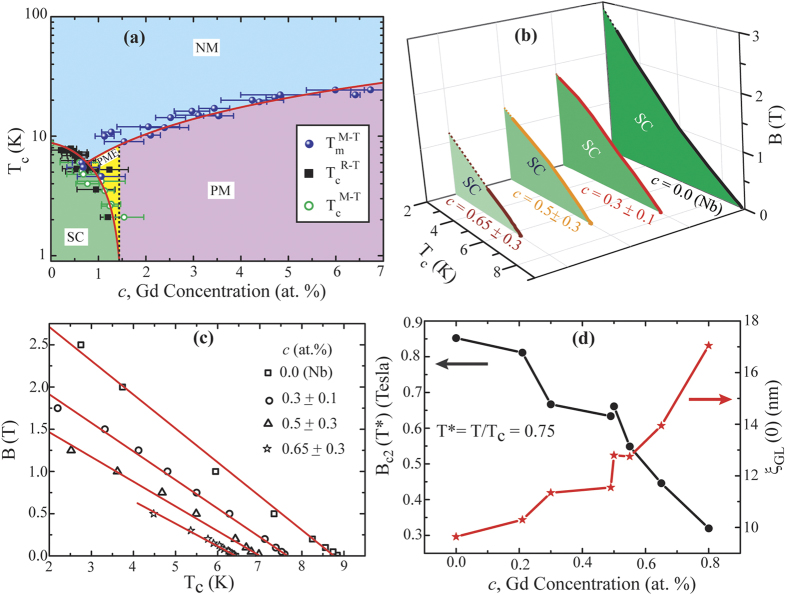
Phase diagram. (**a**) A compositional phase diagram constituted by measuring the transition temperatures for devices with different Gd concentration. The T_c_ values are measured through R-T and DC M-T measurements while the latter also provides the values of magnetic transition temperature T_m_. (**b**) The magnetic (B-T_c_) phase diagram for a few representing samples with different composition. For clarity curves are placed separately along *z*-axis in 3D representation. The solid lines represent the experimental data and the corresponding shaded areas with dark green represent the SC state. The gradient in the color shade indicates the variation in the composition. (**c**) 2-D representation of (B-T_c_) phase diagram for the same devices presented in (b). Scattering points represent the experimental values measured from the field dependence of the R-T characteristics from each sample. The solid red lines are the linear fits. (**d**) Left: Variation of B_c2_(T*) values obtained from the linear fit at T* = T/T_c_ = 0.75 with the Gd concentration. Right: The dependence of the GL coherence length ξ_GL_(0), calculated from the slope of the linear fit for the B-T_c_ data, with *c*. The solid lines are guide to the eye.

Based on the detailed experimental investigations, we have formulated (i) a compositional phase diagram and (ii) a magnetic phase diagram for selected compositions. The dependence of the characteristic transition temperatures on the concentration of magnetic impurities leads to the compositional phase diagram as shown in Fig. 6(a). The black squares and the green circles represent the superconducting critical temperatures measured through R-T and M-T measurements, respectively. The red solid curve (in the left), representing the best fit to the experimental points using the AG theory, illustrates the phase boundary of the SC state (the shaded area in dark green). Above a certain minimum value of the Gd concentration, both the FC and ZFC M-T measurements show a positive magnetization indicating a paramagnetic phase of the CFs under consideration. The paramagnetic transition temperature T_m_ is the temperature related to the onset of the positive magnetization. The T_m_ is observed to depend linearly to the concentration of the magnetic component and the purple spheres in Fig. 6(a) represent the experimental values of T_m_ while the attached red curve in right is a linear fit. The region below this line in the phase diagram indicates the PM phase. However, For a few NbGd samples with *c*-values in the range of 0 < *c* < *c*_cr_, a crossover from paramagnetic to diamagnetic state has been observed in the ZFC M-T measurements while their M-T characteristics in FC condition show positive magnetization down to the measurement limit of 1.8 K [Fig. 4(b)]. This phenomenon is known as the PME. The yellow region, between the SC and the PM states illustrates the region related to the PME. The rest part of the phase diagram obviously represents the NM phase of the CFs. This is noteworthy to mention that the electronic states are very sensitive to the magnetic impurity as revealed in the phase diagram through the appearance of the PME region.

For constructing the magnetic phase diagram we have extracted the T_c_ values from the R-T measurements performed under externally applied magnetic field as previously shown in Fig. 5. For each device with particular *c*-values, we map the B-T_c_ phase diagram. Figure 6(b) contains a set of B-T_c_ phase diagram for a few representative samples with different *c*-values. The shaded areas represent the SC state while the solid lines connect the experimental values. The dotted lines are the extrapolations to the base temperature, 2K. We have used a gradient in the color for the shaded area to indicate the inclusion and variation of the magnetic constituent. The region of superconducting area in the phase diagram decreases with increasing *c* which is consistent with the pair breaking mechanism discussed previously. The experimental data along with their linear fits for the same set of devices are shown in Fig. 6(c). From the GL theory near the T_c_, a linear dependence of T_c_ on the B-field is expected for a dirty superconductor^40^,^41^. We have extracted the B_c2_(T*) value for each device at T* = T/T_c_ = 0.75 from the linear fit and its dependence on *c* is displayed in the left axis of Fig. 6(d). As expected, B_c2_(T*) decreases with increasing *c* since the concentration of the magnetic part and the external magnetic field behave in a complimentary fashion. From the GL theory the coherence length ξ_GL_(0) can be obtained by the slope of the linear fit of the B-T_c_ data as^42^,


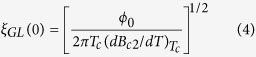


Where, *ϕ*_0_ is the flux quantum. We have calculated the values of ξ_GL_(0) for different Gd concentration in the range between 0 to 0.8 % and the related variation is shown by the red stars in Fig. 6(d). ξ_GL_(0) increases with *c* and this is consistent with the dependence of B_c2_ on *c*. However, with increasing *c* the mean free path gets reduced due to increased scattering and hence the coherence length should also be reduced^42^. Also note that the ξ_GL_(0) for the Nb sample is much lower than that of a pure bulk Nb. This is because the Nb films were grown in the same chamber where NbGd samples were prepared and it is quite likely that our Nb films are in the dirty limit with a low residual resistivity (R_300 K_/R_10 K_ ~ 2.78). Another reason could be the grain size of the Nb films which are in the range of 20–30 nm^22^. The similar value of the ξ_GL_(0) has been reported for Nb with grain sizes ~20 nm^43^. Further, the enhancement of the ξ_GL_(0) with Gd concentration is not only related to the grain size since at this low range of Gd concentration the variation in the grain sizes are not detectable from the AFM studies. The enhancement can be related to the interaction between SC and FM and further study is needed for clear understanding.

Finally, we have demonstrated a pronounced and controlled modulation of the superconducting properties in NbGd based CFs for Gd concentration in the range between 0 to 1.4 at.% by performing M-T and R-T measurements. A critical concentration of Gd ~ 1.3 at.% is found above which superconductivity disappears. Besides, the traces of magnetism with less than 1 at.% Gd in the CFs have been appeared evidently through the PME in M-T measurements and the shouldering effect in R-T measurements. However we do observe a non-monotonous oscillatory dependence of T_c_ on *c* from M-T measurements for the samples with *c* ≥ 1.4 at.%. With high *c*-value, diamagnetic transition can occur partially through any percolating path or due to in-homogeneities mediated by possible Gd clustering. Also the AFM images in Fig. 1(d–f) indicate of possible Gd clustering at high Gd concentration. There could be other effects like the formation of the triplet states originating from the interaction between SC and FM which could lead to an oscillatory behaviour in the T_c_ as reported earlier^20^. Nevertheless, the R-T measurements did not show similar behaviour except for the partial transitions (Figure S3 in the supporting material). Hence we can assume that the non-monotonous behaviour is related to the partial transition.

## Methods

We fabricated NbGd based CFs by co-sputtering of Gd (99.95 %) and Nb (99.99 %) using an ultra-high vacuum (UHV) DC magnetron sputtering system. CFs are grown on Si (100) substrate with a top layer of 300 nm thick thermally oxidized SiO_2_ as the dielectric. The sputtering chamber was evacuated to less than 5 × 10^−9^ Torr before each deposition and the co-sputtering was performed in an Ar (99.9999 % purity) environment at about 3 x 10^-3^ mBar. The deposition rates for Nb and Gd were 3 Å/sec and 1–2 Å/sec, respectively. Apart from the determination of the sputtering rate, we had performed an extensive optimization process with respect to the relative position of the samples from the sputtering targets and the open area for the shutter of Gd target in order to obtain a gradient in the Gd concentration with respect to the relative position of the samples from the targets. Using this technique we have been able to manufacture samples with varying Gd concentration but keeping the Nb part unaltered from one single sputtering run. For different deposition runs we tried to maintain the relevant parameters very closely so that the results could be comparable from samples fabricated in different sputtering runs. On this context, we prepared 2 batches with thicknesses ~60 nm and ~110 nm, respectively, and each batch contained 6 devices with variation in Gd concentration. Devices from both the batches follow the AG theory independently and the related *c*_cr_ values remained very close (see the supporting material). This confirms that the devices prepared from different batches with similar conditions can be comparable.

We mainly prepared two types of samples, namely, CFs with dimensions 3.5 mm × 3.5 mm × 100 nm for DC M-T measurements and NbGd based patterned devices for transport studies. CFs, studied in this report, were grown at room temperature with thickness varying in the range of 50–110 nm. A Si capping layer of about 10 nm thickness was sputtered on top of the CFs for both types of samples to avoid any oxidation while exposed to the atmosphere. We employed shadow mask to fabricate multi-terminal devices for conventional 4-terminal transport measurements. Current and voltage leads were designed with Au (100 nm)/Ti (20 nm) layers followed by the fabrication of about 100 micron wide and about 1300 micron long channel of NbGd based CFs. Careful measurements were done for the estimation of the concentration by energy dispersive spectroscopy (EDS) analysis using a field emission scanning electron microscope by Zeiss. For each sample, EDS measurements were performed at 5–10 different places and the average value was considered for the analysis along with the uncertainty range estimated from the measured composition at different places. Structural characterization of CFs with varying *c* was done by grazing incidence X-ray diffraction (GIXRD) using a θ-2θ x-ray diffractometer (Philips X’pert pro X-ray diffractometer) with Cu-k_α_ radiation operating at 40 kV and 20 mA and the morphological studies were performed by an atomic force microscopy (AFM). A SQUID magnetometer from Quantum Design was used to perform the M-T measurements. The transport measurements were carried out in a Physical Properties Measurement System (PPMS) by Quantum Design. The devices were mounted on a puck and soldered for the electrical measurements.

## Additional Information

**How to cite this article**: Bawa, A. *et al.* Ultrasensitive interplay between ferromagnetism and superconductivity in NbGd composite thin films. *Sci. Rep.* **6**, 18689; doi: 10.1038/srep18689 (2016).

## Supplementary Material

Supplementary Information

## References

[b1] Buzdin A. I. Proximity effects in superconductor-ferromagnet heterostructures. Rev. Mod. Phys. 77, 935 (2005).

[b2] Aladyshkin A. Y., Silhanek A. V., Gillijns W. & Moshchalkov V. V. Nucleation of superconductivity and vortex matter in superconductor-ferromagnet hybrids. Supercond. Sci. Technol. 22, 053001 (2009).

[b3] Wei T. C., Pekker D., Rogachev A., Bezryadin A. & Goldbart P. M. Enhancing superconductivity: Magnetic impurities and their quenching by magnetic fields. Europhys. Lett. 75, 943 (2006).

[b4] Stamopoulos D., Pissas M., Karanasos V., Niarchos D. & Panagiotopoulos I. Influence of randomly distributed magnetic nanoparticles on surface superconductivity in Nb films. Phys. Rev. B 70, 054512 (2004).

[b5] Yang Z., Lange M., Volodin A., Szymczak R. & Moshchalkov V. V. Domain-wall superconductivity in superconductor-ferromagnet hybrids. Nat. Mater. 3, 793 (2004). 15467724 10.1038/nmat1222

[b6] Linder J. & Halterman K. Superconducting spintronics with magnetic domain walls. Phys. Rev. B 90, 104502 (2014).

[b7] Stamopoulos D., Manios E., Pissas M. & Niarchos D. Modulation of the properties of a low-T_c_ superconductor by anisotropic ferromagnetic particles. Physica C 437–38, 289 (2006).

[b8] Palau A. *et al.* Hysteretic Vortex Pinning in Superconductor-Ferromagnet Nanocomposites. Phys. Rev. Lett. 98, 117003 (2007). 17501078 10.1103/PhysRevLett.98.117003

[b9] Togoulev P. N., Suleimanov N. M. & Conder K. Pinning enhancement in MgB_2_-magnetic particles composites. Physica C 450, 45 (2006).

[b10] Bergeret F. S., Volkov A. F. & Efetov K. B. Odd triplet superconductivity and related phenomena in superconductor-ferromagnet structures. Rev. Mod. Phys. 77, 1321 (2005). 10.1103/PhysRevLett.90.117006 12688960

[b11] Eschrig M. Spin-polarized supercurrents for spintronics. Phys. Today 64, 43–49 (2011). 10.1088/0034-4885/78/10/104501 26397456

[b12] Strelniker Y. M., Frydman A. & Havlin S. Percolation model for the superconductor-insulator transition in granular films. Phys. Rev. B 76, 224528 (2007).

[b13] Liu X., Panguluri R. P., Huang Z.-F. & Nadgorny B. Double Percolation Transition in Superconductor-Ferromagnet Nanocomposites. Phys. Rev. Lett. 104, 035701 (2010). 20366657 10.1103/PhysRevLett.104.035701

[b14] Matthias B. T., Suhl H. & Corenzwit E. Spin Exchange in Superconductors. Phys. Rev. Lett. 1, 92 (1958).

[b15] Kim H. *et al.* Effect of magnetic Gd impurities on the superconducting state of amorphous Mo-Ge thin films with different thickness and morphology. Phys. Rev. B 86, 024518 (2012).

[b16] Strunk C., Sürgers C., Paschen U. & Löhneysen H. v. Superconductivity in layered Nb/Gd films. Phys. Rev. B 49, 4053 (1994). 10.1103/physrevb.49.4053 10011303

[b17] Koch C. C. & Love G. R. Superconductivity in Niobium Containing Ferromagnetic Gadolinium or Paramagnetic Yttrium Dispersions. J. Appl. Phys. 40, 3582 (1969).

[b18] Scholten P. D. & Moulton W. G. Effect of ion-implanted Gd on the superconducting properties of thin Nb films. Phys. Rev. B 15, 1318 (1977).

[b19] Strunk C., Paschen U., Surgers C. & Vonlohneysen H. Pair-breaking mechanisms in Nb/Gd/Nb films. Physica B 194, 2403 (1994).

[b20] Jiang J. S., Davidović D., Reich D. H. & Chien C. L. Oscillatory Superconducting Transition Temperature in Nb/Gd Multilayers. Phys. Rev. Lett. 74, 314 (1995). 10058357 10.1103/PhysRevLett.74.314

[b21] Strunk C., Surgers C., Rohberg K. & Vonlohneysen H. Transition-temperature and critical fields of Nb/Gd layers Physica B 194, 2405 (1994).

[b22] Bawa A., Jha R. & Sahoo S. Tailoring phase slip events through magnetic doping in superconductor-ferromagnet composite films. Sci. Rep. 5, 13459 (2015). 26304594 10.1038/srep13459 PMC4548245

[b23] Abrikosov A. A. & Gor’kov L. P. Contribution to the theory of superconducting alloys with paramagnetic impurities. Sov. Phys. JETP 12, 1254 (1961).

[b24] Sommer R. L., Xiao J. Q. & Chien C. L. Magnetic and magneto-transport properties of metastable Gd_x_Nb_1-x_ alloys. IEEE Trans. Magn. 34, 1135 (1998).

[b25] Li M. S. Paramagnetic Meissner effect and related dynamical phenomena. Phys. Rep. 376, 133 (2003).

[b26] Braunisch W. *et al.* Paramagnetic Meissner effect in high-temperature superconductors. Phys. Rev. B 48, 4030 (1993). 10.1103/physrevb.48.4030 10008853

[b27] Braunisch W. *et al.* Paramagnetic Meissner effect in Bi high-temperature superconductors. Phys. Rev. Lett. 68, 1908 (1992). 10045251 10.1103/PhysRevLett.68.1908

[b28] Kim Y.-J. & Overhauser A. W. Theory of impure superconductors: Anderson versus Abrikosov and Gor’kov. Phys. Rev. B 47, 8025 (1993). 10.1103/physrevb.47.8025 10004812

[b29] Powell B. J. & McKenzie R. H. Dependence of the superconducting transition temperature of organic molecular crystals on intrinsically nonmagnetic disorder: A signature of either unconventional superconductivity or the atypical formation of magnetic moments. Phys. Rev. B 69, 024519 (2004).

[b30] Fang Y., Yazici D., White B. D. & Maple M. B. Enhancement of superconductivity in La_1−x_Sm_x_O_0.5_F_0.5_BiS_2_. Phys. Rev. B 91, 064510 (2015).

[b31] Thompson D. J., Minhaj M. S. M., Wenger L. E. & Chen J. T. Observation of Paramagnetic Meissner Effect in Niobium Disks. Phys. Rev. Lett. 75, 529 (1995). 10060044 10.1103/PhysRevLett.75.529

[b32] Yuan S., Ren L. & Li F. Paramagnetic Meissner effect in Pb nanowire arrays. Phys. Rev. B 69, 092509 (2004).

[b33] Papadopoulou E. L., Nordblad P., Svedlindh P., Schöneberger R. & Gross R. Magnetic Aging in Bi_2_Sr_2_CaCu_2_O_8_ Displaying the Paramagnetic Meissner Effect. Phys. Rev. Lett. 82, 173 (1999).

[b34] Das P. *et al.* Surface superconductivity, positive field cooled magnetization, and peak-effect phenomenon observed in a spherical single crystal of niobium. Phys. Rev. B 78, 214504 (2008).

[b35] Xing Y. T., Micklitz H., Baggio-Saitovitch E. & Rappoport T. G. Controlled switching between paramagnetic and diamagnetic Meissner effects in superconductor-ferromagnet Pb-Co nanocomposites. Phys. Rev. B 80, 224505 (2009).

[b36] López de la Torre M. A. *et al.* Paramagnetic Meissner effect inYBa_2_Cu_3_O_7_/La_0.7_Ca_0.3_MnO_3_ superlattices. Phys. Rev. B 73, 052503 (2006).

[b37] Moshchalkov V. V., Qiu X. G. & Bruyndoncx V. Paramagnetic Meissner effect from the self-consistent solution of the Ginzburg-Landau equations. Phys. Rev. B 55, 11793 (1997).

[b38] Zharkov G. F. Paramagnetic Meissner effect in superconductors from self-consistent solution of Ginzburg-Landau equations. Phys. Rev. B 63, 214502 (2001).

[b39] Xing Y. T. *et al.* Spontaneous vortex phases in superconductor-ferromagnet Pb-Co nanocomposite films. Phys. Rev. B 78, 224524 (2008).

[b40] Vicent J. L., Hillenius S. J. & Coleman R. V. Critical-Field Enhancement and Reduced Dimensionality in Superconducting Layer Compounds. Phys. Rev. Lett. 44, 892 (1980).

[b41] Tinkham M. Introduction to Superconductivity. 2nd edn, (McGraw-Hill, 1996).

[b42] Orlando T. P., McNiff E. J., Foner S. & Beasley M. R. Critical fields, Pauli paramagnetic limiting, and material parameters of Nb_3_Sn and V_3_Si. Phy. Rev. B 19, 4545 (1979).

[b43] Bose S., Raychaudhuri P., Banerjee R. & Ayyub P. Upper critical field in nanostructured Nb: Competing effects of the reduction in density of states and the mean free path. Phys. Rev. B 74, 224502 (2006).

