# Supplementary material for: Ultrasensitive interplay between ferromagnetism and superconductivity in NbGd composite thin films
Source: Sci Rep. 2016 Jan 4;6:18689. doi: 10.1038/srep18689 (PMC4698659; doi:10.1038/srep18689)
Supplement: Supplementary Information [file srep18689-s1.pdf]

Supporting Material

Ultrasensitive interplay between ferromagnetism and superconductivity in NbGd composite thin films

*Ambika Bawa[1], Anurag Gupta[1], Sandeep Singh[2], V.P.S. Awana[1] & Sangeeta Sahoo[1]**

[1]Quantum Phenomena & Applications, CSIR-National Physical Laboratory, Council of Scientific and Industrial Research, Dr. K. S. Krishnan Marg, New Delhi, India- 110012

[2]Sophisticated and Analytical Instrumentation, CSIR-National Physical Laboratory, Council of Scientific and Industrial Research, Dr. K. S. Krishnan Marg, New Delhi, India- 110012

**Correspondence and requests for materials should be addressed to S.S.(sahoos@nplindia.org)*

Fabrication of NbGd based composite films (CFs):

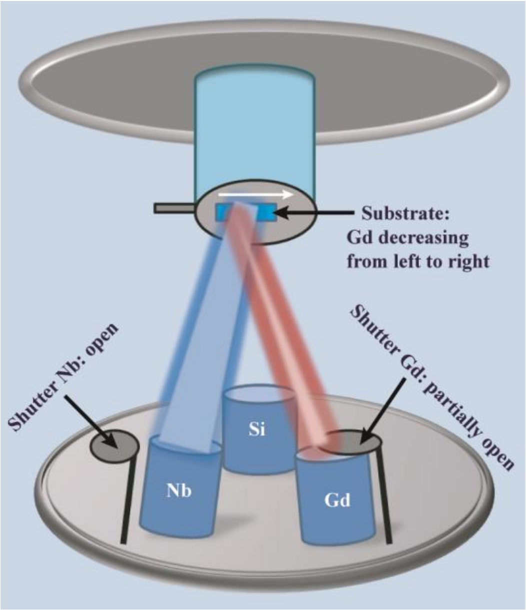

Figure S1: Schematic presentation of sample fabrication

We have prepared NbGd based CFs with variations in Gd concentration while keeping Nb concentration almost unaltered throughout the substrate. Figure S1 represents schematically the process during a sputtering run. The sample is placed in such a way that the deposition rate for Nb is uniform for a wide area of the sample while the deposition rate for Gd is varied by the shutter position and the relative position of the sample. The resultant film provides a gradient in Gd concentration along the long side of the strip as indicated by the white arrow. By this way we usually prepare 6 devices at one single run for transport measurement. For the magnetization measurements we use a long Si strip at the beginning and after the sputtering we cut into several pieces of 3.5 mm x 3.5 mm dimensions. We can place more than one strip in parallel and close to each other.

Note that the differences in the *c*-values mentioned in the main manuscript are noticeably small with relatively higher uncertainties which most likely come from the bigger area of the sample with more probable non-uniform distribution of the magnetic particles. However, as mentioned in the experimental

section, we paid careful attention towards determining the composition by energy dispersive spectroscopy (EDS) at many places and the relative uncertainties are provided as the error bars accompanied with the average values. Further, the order (increasing and/decreasing) in the *c* values for device to device are consistent with their position during a sputtering run. Also, before the fabrication of the devices used for this study, we had performed an extensive and rigorous optimization process in order to deal with the relative Gd concentration for sample to sample with their specific position inside the sputtering chamber while keeping the Nb concentration almost unaltered.

Robustness of the sample properties:

In order to compare the samples prepared in different runs we have investigated the modulation of the superconducting properties with Gd concentration for devices made in individual runs and also with variation in the film thickness. Figure S2 represents the variation of the normalized transition temperature with Gd concentration for two sets of devices being fabricated in two separate sputtering runs performed under similar growth conditions. The first set of devices [Figure S2 (a)] are having thicknesses ~60 nm and for the second batch [Figure S2 (a)] the same is about 110 nm. Both the sets independently follow the AG theory as indicated by the red curves [the fits using equation (3) in the main manuscript]. And the critical concentration (c_{cr}) values are also close. This clearly states that within this range of thickness values one can have comparable results from the devices grown at different sputtering runs. To get a more accurate value of c_{cr}, we have collected the data from many more devices grown at different sputtering runs and the results are presented in the manuscript.

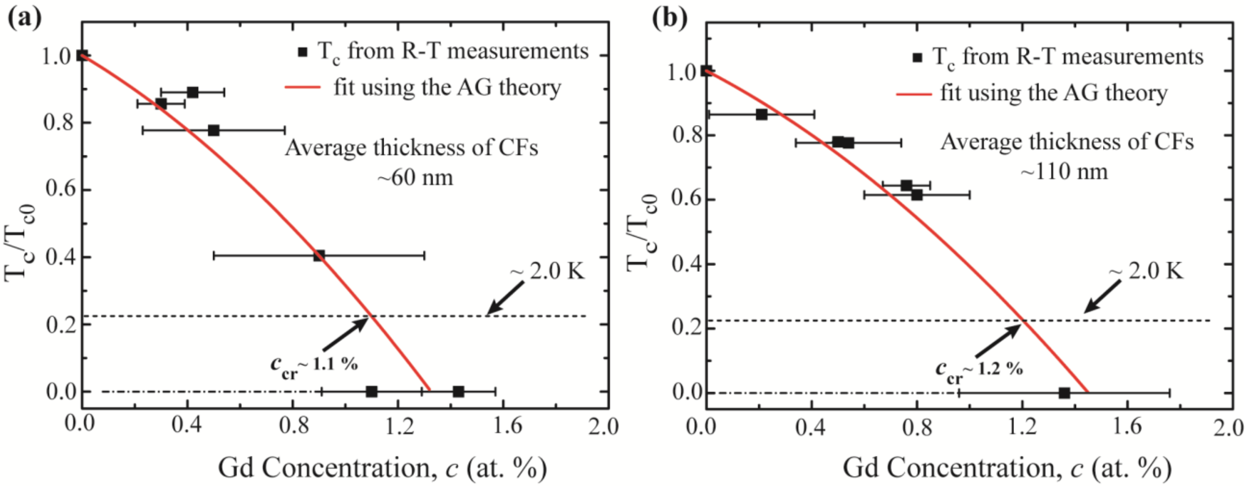

Figure S2: Variation of T_c with Gd concentration for two different batches of samples. T_c/T_{c0} vs. *c* for the devices with average thickness (a) ~ 60 nm and (b) ~ 110 nm. The T_c is extracted from R-T measurements. The black squares are the measured values and the red solid curves represent fits using the AG theory [equation (3) in the manuscript]. The dotted lines represent the temperature limit of our measurement setup.

Partial transition for the devices with higher values of *c* from the R-T measurements:

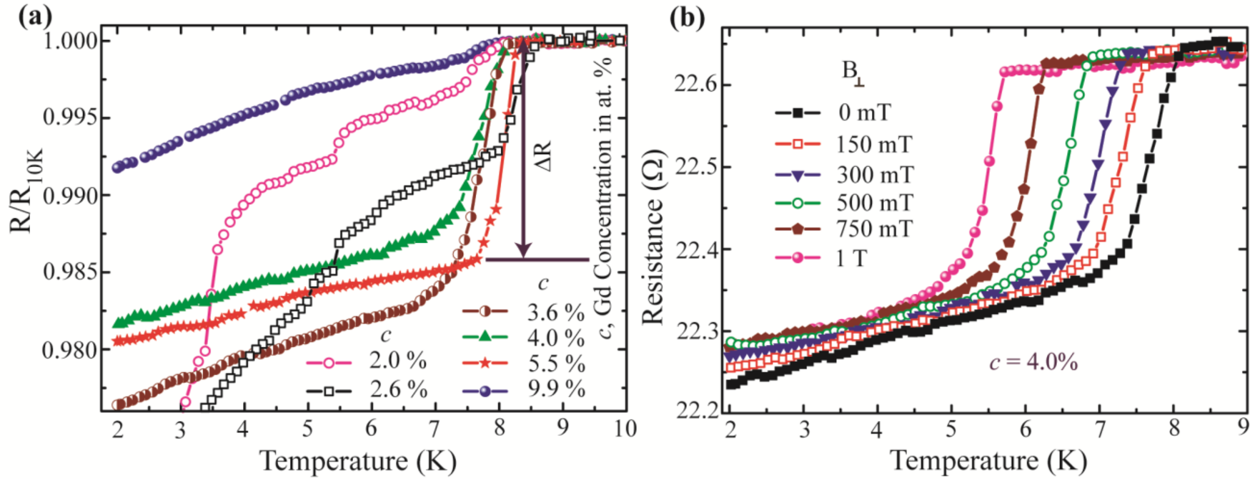

Figure S3: R-T measurements for the devices with higher Gd concentration. (a) A set of R-T characteristics at zero external magnetic field. ΔR indicates the resistance drop. (b) The data of R-T measurements performed under an external magnetic field for a representative sample with *c* = 4%

As indicated in the manuscript, we do observe a resistance drop (ΔR) for the samples with high Gd content. In this case, the resistance does not reach its zero value but there is a drop which appears in the R-T characteristics evidently. We call theses transitions as the partial/incomplete transitions and most likely these transitions are local event and not through the bulk of the sample. To understand the origin of these transitions and to address whether they are of superconducting origin, we have measured the R-T dependence under an external magnetic field and we have shown the same in Figure S3(b) for one representative sample (c = 4%). A clear resistance drop appears with a lower temperature shift for higher B-field. This is very similar to what is expected for superconducting transition and hence we call them as the partial/incomplete transitions.

Variation of the magnetic transition temperature T_m with Gd concentration for samples prepared in a single deposition run:

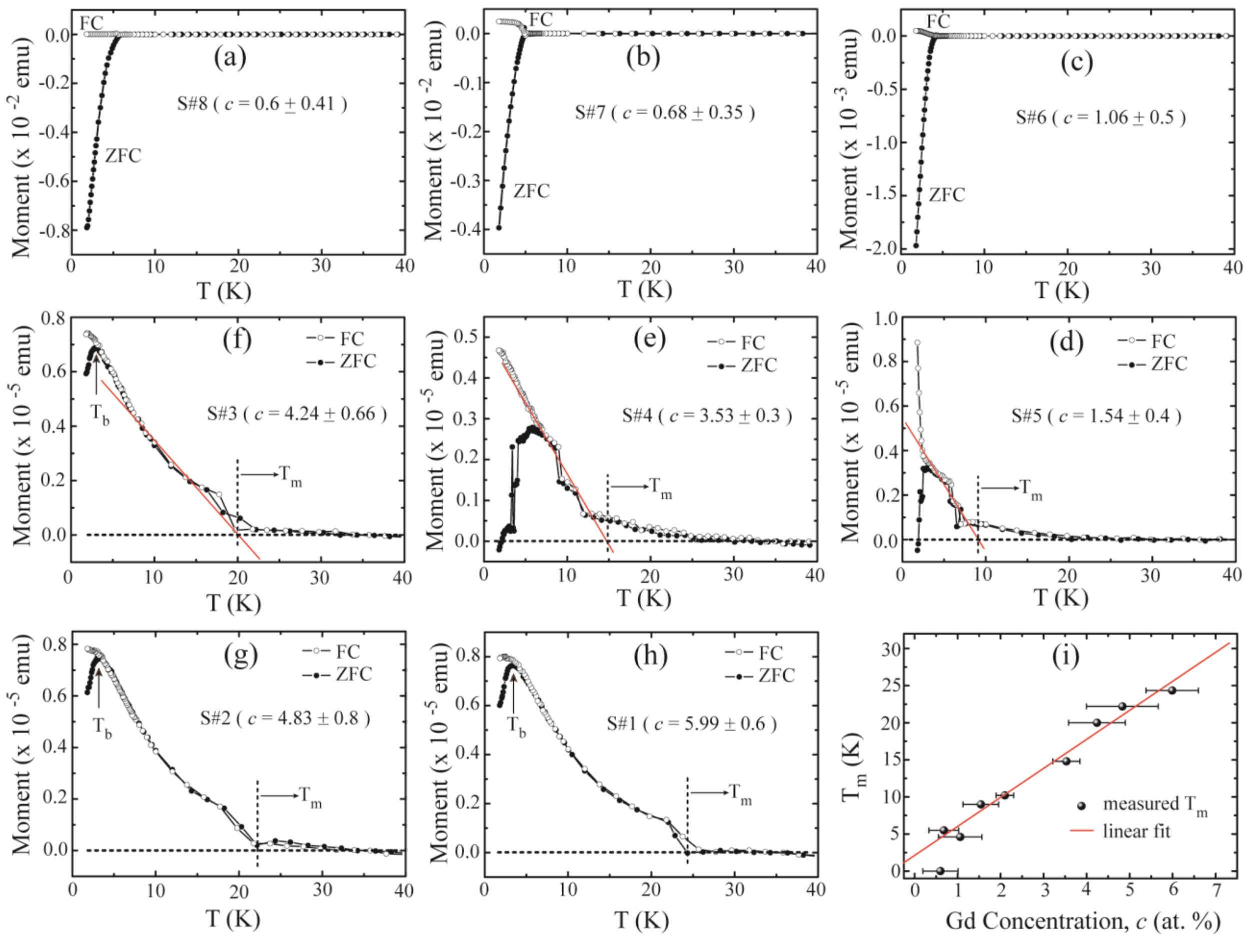

Figure S4: DC M-T measurements for a set of samples grown at single sputtering run. The transition from diamagnetic to paramagnetic state with increasing Gd concentration is evident from (a) to (h). The magnetic transition temperature T_m is shown in (d)-(h). The red lines in (d)-(f) represent the slope of the positive magnetization and the intersect points of theses slopes to the zero-magnetization provide the values of T_m. With higher Gd incorporation the blocking temperature T_b appears in the M-T measurements. (i) Variation of T_m with Gd concentration. The spherical scattering points represent the measured T_m values and the red line is a linear fit.

The DC M-T measurements in both FC and ZFC conditions for a set of samples prepared in a single sputtering run are shown in Figure S3. The sample S#8 was taken from the extreme right of a sample strip while the sample S#1 was placed at the extreme left (see Figure S1 for reference). The order of Gd concentration is decreasing from S#1 to S#2 to S#3 and so on up to S#8. The transition from a diamagnetic state to a paramagnetic state with increasing Gd is evident from the figure. The red lines in (d)-(f) of Figure S3 closely follow the magnetization curve in the paramagnetic state. The dotted black lines indicate the zero magnetization state. We have extracted the T_m values from the intersect points of these red lines to the zero-magnetization line. The samples, with relatively higher Gd concentration shown in (g)-(h) of Figure S3, switches sharply to the positive magnetization state and the switching temperature is considered as the magnetic transition temperature T_m. Finally, we have plotted the T_m values for this set of samples with Gd concentration in Figure S3 (i) and the dependence is linear as it is evident by the linear fit [the red line in Figure S3 (i)].

From the M-T data presented in Figure S4, we observe several interesting phenomena, namely, (i) the onset of positive magnetization in ZFC changes from a gradual increase [(d) & (e)] to a relatively sharp switching [(f) - (h)] for increasing c-values, (ii) at the peak (T_b) of ZFC, FC and ZFC starts to deviate, (iii) at c ~ 1%, the T_m value deviates from the linear behaviour [Figure S4(i)]. The first two points can be understood in the light of superparamagnetism in the Gd particles. From the AFM studies we have got the indication that the particle size increases with Gd concentration. The average particle size for c = 5 % is about 50-60 nm [Figure 1(d) in the main text] and the same for c = 9 % is about 130-140 nm [Figure 1(e) in the main text]. Hence for the lower range of c- values Gd particles can be treated as superparamagnetic in nature. At relative low Gd concentration the thermal fluctuation can have stronger influence on the particles due to their smaller size and hence positive the magnetization starts to appear smoothly [(d) & (e)] whereas, the thermal fluctuation is supposed to have less impact on the larger particles and hence we observe a relatively sharp transition [(d) & (e)]. Also at T_b the deviation of the ZFC and FC curves indicate of freezing of the magnetic moments implying the superparamagnetic nature of the Gd particles. In this case, T_m behaves as the Curies temperature and the T_b is related to the blocking temperature. The third point, the deviation in T_m from the linear behaviour is not clear and it could be related to the influence of the superconducting Nb matrix on the magnetic ordering. However, we feel that we need to perform more experiments like the field dependent magnetization measurement along with their detailed structural analysis using high resolution TEM to have a clear understanding of the magnetic behaviour on the particle size and the clustering effect for high Gd concentration.
